# Renal amyloid‐A amyloidosis in cats: Characterization of proteinuria and biomarker discovery, and associations with kidney histology

**DOI:** 10.1111/jvim.16920

**Published:** 2023-11-22

**Authors:** Carlo Palizzotto, Felippo Ferri, Carolina Callegari, Francesco Rossi, Marcello Manfredi, Laura Carcangiu, Gabriele Gerardi, Silvia Ferro, Laura Cavicchioli, Elizabeth Müller, Marco Weiss, Anne‐Catherine Vogt, Francesca Lavatelli, Stefano Ricagno, Karyl Hurley, Eric Zini

**Affiliations:** ^1^ AniCura Istituto Veterinario Novara Granozzo con Monticello NO Italy; ^2^ Department of Animal Medicine, Production and Health University of Padova Legnaro PD Italy; ^3^ Studio Veterinario Associato Vet2Vet di Ferri e Porporato Orbassano TO Italy; ^4^ Department of Translational Medicine University of Piemonte Orientale Novara Italy; ^5^ Department of Comparative Biomedicine and Food Science University of Padova Legnaro PD Italy; ^6^ Laboklin, Laboratory for Clinical Diagnostics Bad Kissingen Germany; ^7^ Department of Rheumatology and Immunology University Hospital Bern Bern Switzerland; ^8^ Department of BioMedical Research University of Bern Bern Switzerland; ^9^ Graduate School for Cellular and Biomedical Sciences (GCB) University of Bern Bern Switzerland; ^10^ Department of Molecular Medicine University of Pavia Pavia Italy; ^11^ Institute of Molecular and Translational Cardiology IRCCS Policlinico San Donato Milan Italy; ^12^ Department of Biosciences Università degli Studi di Milano Milan Italy; ^13^ Mars Veterinary Health McLean Virginia USA; ^14^ Clinic for Small Animal Internal Medicine, Vetsuisse Faculty University of Zurich Zurich Switzerland

**Keywords:** chronic kidney disease, diagnosis, feline, fibrils

## Abstract

**Background:**

Amyloid A (AA) amyloidosis is a protein misfolding disease arising from serum amyloid A (SAA). Systemic AA amyloidosis recently was shown to have a high prevalence in shelter cats in Italy and was associated with azotemia and proteinuria.

**Objectives:**

Investigate urine protein profiles and diagnostic biomarkers in cats with renal AA amyloidosis.

**Animals:**

Twenty‐nine shelter cats.

**Methods:**

Case‐control study. Cats with renal proteinuria that died or were euthanized between 2018 and 2021 with available necropsy kidney, liver and spleen samples, and with surplus urine collected within 30 days before death, were included. Histology was used to characterize renal damage and amyloid amount and distribution; immunohistochemistry was used to confirm AA amyloidosis. Urine protein‐to‐creatinine (UPC) and urine amyloid A‐to‐creatinine (UAAC) ratios were calculated, and sodium dodecyl sulfate‐agarose gel electrophoresis (SDS‐AGE) and liquid chromatography‐mass spectrometry (LC‐MS) of proteins were performed.

**Results:**

Twenty‐nine cats were included. Nineteen had AA amyloidosis with renal involvement. Cats with AA amyloidosis had a higher UPC (median, 3.9; range, 0.6‐12.7 vs 1.5; 0.6‐3.1; *P* = .03) and UAAC ratios (median, 7.18 × 10^−3^; range, 23 × 10^−3^‐21.29 × 10^−3^ vs 1.26 × 10^−3^; 0.21 × 10^−3^‐6.33 × 10^−3^; *P* = .04) than unaffected cats. The SDS‐AGE identified mixed‐type proteinuria in 89.4% of cats with AA amyloidosis and in 55.6% without AA amyloidosis (*P* = .57). The LC‐MS identified 63 potential biomarkers associated with AA amyloidosis (*P* < .05). Among these, urine apolipoprotein C‐III was higher in cats with AA amyloidosis (median, 1.38 × 10^7^; range, 1.85 × 10^5^‐5.29 × 10^7^ vs 1.76 × 10^6^; 0.0 × 10^0^‐1.38 × 10^7^; *P* = .01). In the kidney, AA‐amyloidosis was associated with glomerulosclerosis (*P* = .02) and interstitial fibrosis (*P* = .05).

**Conclusions and Clinical Importance:**

Renal AA amyloidosis is associated with kidney lesions, increased proteinuria and increased urine excretion of SAA in shelter cats. Additional studies are needed to characterize the role of lipid transport proteins in the urine of affected cats.

**Abbreviations**
AA: amyloid‐A
Apo: apolipoprotein
CKD: chronic kidney diseases
DSH: domestic shorthair
FeLV: feline leukemia virus
FIP: feline infectious peritonitis
FIV: feline immunodeficiency virus
HMW: high molecular weight
IRIS: International Renal Interest Society
LC‐MS: liquid chromatography‐mass spectrometry
LMW: low molecular weight
SAA: serum amyloid‐A
SDMA: symmetric dimethylarginine
SDS‐AGE: sodium dodecyl sulfate‐agarose gel electrophoresis
UAAC: urine amyloid A‐to‐creatinine
UPC: urine protein‐to‐creatinine
USG: urine specific gravity

## 1 INTRODUCTION

Amyloidosis is a group of diseases characterized by the extracellular deposition of insoluble proteins. Its classification relies upon identification of the type of misfolded protein and the organ involved.^1^ Currently, in cats 4 different forms of amyloidosis are described: amyloid‐producing odontogenic tumors, amyloid A (AA), light chain and pancreatic islet amyloidosis.^2^, ^3^, ^4^, ^5^, ^6^ Amyloidosis of the AA type, which arises from the acute phase protein serum amyloid A (SAA) produced by hepatocytes during inflammation, is the most frequent in this species.^2^, ^3^, ^7^ Serum amyloid A protein can undergo conformational changes into ß‐sheet structures and deposit as insoluble amyloid fibrils in different tissues, such as the spleen, liver, and kidney.^2^, ^3^, ^7^

In cats, AA amyloidosis is reported as familial in Abyssinian and Siamese breeds.^8^, ^9^, ^10^, ^11^ In domestic shorthair (DSH) cats, the disease is rare but has been reported in association with liver rupture, pulmonary hemorrhage, and feline immunodeficiency virus.^12^, ^13^, ^14^, ^15^ Recently, an extremely high prevalence of AA amyloidosis, ranging from 52% to 73%, was reported in domestic shorthair cats from 3 Italian shelters and its fibril structure was fully characterized.^16^, ^17^

In affected humans, nonfamilial AA amyloidosis occurs as a sequela of chronic inflammatory diseases, leading to proteinuria and end‐stage renal failure.^18^ In a previous study in a cat shelter with high prevalence of AA amyloidosis, a score derived from abnormal laboratory variables was used to assess the severity of disease.^19^ Cats with AA amyloidosis had higher scores than those without, suggesting that inflammatory diseases, as in humans, play a permissive role in the deposition of amyloid fibrils.^19^, ^20^ Furthermore, 79% of shelter cats with AA amyloidosis had renal amyloid deposit, and proteinuria or azotemia was common.^20^

Renal AA amyloidosis in cats generally is suspected in case of severe proteinuria, but it requires tissue histopathology and rarely is diagnosed antemortem. In Abyssinian cats with familial AA amyloidosis, the SAA concentration is increased but the wide overlap between affected and nonaffected animals does not allow its use as a disease biomarker.^21^ In a recent study in Abyssinian cats, those from a breeding facility with high prevalence of AA amyloidosis had higher urine amyloid A‐to‐creatinine (UAAC) ratios in comparison to those from a breeding facility with low prevalence, and the test was proposed as a potential biomarker.^22^

In the last decade, proteomics‐based technologies have played a pivotal role in the characterization of amyloid structure and related proteins in humans and animals.^23^, ^24^, ^25^ In 2 recent studies, apolipoprotein (Apo) E, Apo A‐I, and Apo I‐V were identified in renal samples from Abyssinian, Siamese and DSH cats with AA amyloidosis.^26^, ^27^ To the best of our knowledge, proteomics analysis of urine samples in cats with AA amyloidosis has not been carried out. Thus, our aims were to characterize proteinuria and identify potential diagnostic biomarkers in shelter cats with renal AA amyloidosis, including urine proteomics, and to investigate the relationship between histologic features of fibril deposition in the kidneys and proteinuria.

## 2 MATERIALS AND METHODS

### 2.1 Study design

Our was a retrospective case‐control study. Cats from shelters located in northwest Italy that died or were euthanized because of worsening clinical condition from January 2018 to July 2021 were included. All recruited cats had been enrolled previously in a prospective study on the prevalence of AA amyloidosis in shelter cats.^16^ Signed informed consent was obtained from the owners of the shelters before use of stored samples for the study. Inclusion criteria were necropsy collection of kidney, spleen, and liver within 6 hours after death, proteinuria defined as urine protein‐to‐creatinine (UPC) ratio >0.4, and surplus urine stored at −80°C from samples collected within 30 days before death. Additionally, cases were included if cats with amyloidosis had fibrils documented in the kidney; cases with amyloid in the liver, spleen or both but not in the kidney were excluded.

### 2.2 Clinical and laboratory data

Information on sex, age, breed, duration of stay in the shelter, clinical and laboratory data, including serum creatinine, urea, symmetric dimethylarginine (SDMA) and phosphate concentrations and urinalysis, as well as cause of death if available, were retrieved from medical records. Blood work and urinalyses were performed at IDEXX Laboratories Day Lab (Novara, Italy). As is routine at the authors' institution, urine samples were collected by ultrasound‐guided cystocentesis during diagnostic evaluation. Bacterial culture was performed in cats with clinical signs of lower urinary tract disease, increased serum creatinine concentration or urine sediment suggestive of inflammation. Chronic kidney disease (CKD) was diagnosed and classified according to the International Renal Interest Society (IRIS) guidelines.^28^ Cases with serum creatinine concentration <1.6 mg/dL and SDMA concentration <18 μg/dL were IRIS CKD stage I, those with serum creatinine concentration 1.6‐2.8 mg/dL and serum SDMA concentration 18‐25 stage II, those with serum creatinine concentration 2.9‐5.0 mg/dL and serum SDMA concentration 26‐38 μg/dL stage III, and those with creatinine >5.0 mg/dL and SDMA >38 μg/dL stage IV.

### 2.3 Urinalysis

Surplus urine was thawed and divided into 2 aliquots; the first 250 μL was used for liquid chromatography‐mass spectrometry (LC‐MS) and the second 400 μL for urine specific gravity (USG), UPC ratio, sodium dodecyl sulfate‐agarose gel electrophoresis (SDS‐AGE) and to determine the UAAC ratio. Urine specific gravity, UPC ratio and SDS‐AGE were performed in a commercial laboratory (Laboklin, Bad Kissingen, Germany) whereas LC‐MS was performed in a research facility (University of Piemonte Orientale, Novara, Italy).

### 2.4 UPC ratio and UAAC ratio

Proteins, creatinine, and SAA were measured in native cat urine using a Cobas 8000 instrument (Hitachi, Mannheim, Germany). For urine proteins, the reagent TPUC3 (Roche Diagnostics, Mannheim, Germany) was used and for urine creatinine the reagent CREJ2 (Roche Diagnostics) was utilized. To measure SAA, a turbidimetric immunoassay (Eiken Chemical, Tokyo, Japan) was used with intraassay and interassay coefficients of variations in cat urine of 9.7% and 12.1%, respectively. For spike recovery, different amounts of SAA were added to cat urine at the original concentration of 41.7 μg/mL; the recovery rate was 96.7%‐105.7%. Linearity of dilutions was 76.5%‐105.7%.

### 2.5 SDS‐AGE of urine proteins

Electrophoresis was performed with the Hydragel 5 test (Sebia, Fulda, Germany) using the Hydrasys 2 machine (Sebia). Twenty microliters of diluent were mixed with 80 μL of native urine and vortexed for 5 seconds. Five microliters of treated urine then was applied to the starting well of the agarose gel. The gel was processed by the machine using the following principle: in an excess of an anionic detergent, SDS and proteins are converted into SDS‐protein complexes. In these complexes, all proteins assume the same conformation and the same negative charge per mass unit. In the agarose gel, they separate according to their molecular weight by electrophoresis.^26^ Proteinuria was classified as glomerular (high molecular weight, HMW), tubular (low molecular weight, LMW), or mixed, and a semiquantitative score was applied to define each class as absent, mild, or moderate to severe.^29^

### 2.6 LC‐MC of urine proteins

Thawed urine samples were centrifuged at 1000 rpm for 10 min and the supernatant was separated. For sample preparation, 250 μL of each urine supernatant was precipitated with ethanol by adding 5 volumes of 100% ethanol (1250 μL) followed by 5 minutes of stirring on a rotator mixer, and finally precipitation was completed with overnight incubation at −20°C. The next day, samples were centrifuged at 12 000 g for 15 min, the supernatant was discarded, and the pellet was washed with 1 mL of 70% ethanol. Samples were centrifuged a second time at 12 000 g for 5 min; the supernatant again was discarded and the pellet air‐dried. The pellets were resuspended in 20 μL of 8 M urea/100 mM ammonium bicarbonate and the Bradford assay (Bio‐Rad, Hercules, CA) was used to determine protein concentration. Eighty micrograms of protein then was subjected to in‐solution digestion. Briefly, samples were denatured with trifluoroethyl alcohol (Sigma‐Aldrich, St. Louis, MO), then reduced with 200 mM dithiothreitol, alkylated with 200 mM iodoacetamide, and subjected to overnight digestion with 2 μg of trypsin (Sigma‐Aldrich). The next day, the peptide digests were desalted on a Discovery DSC‐18 solid phase extraction (SPE) 96‐well plate (25 mg/well; Sigma‐Aldrich). Desalted samples were vacuum‐evaporated and reconstituted in 0.1% formic acid for subsequent analysis. The digested peptides were analyzed using ultra‐high performance liquid chromatography (Vanquish system; Thermo Scientific, Rodano, Italy) coupled with an Orbitrap Q‐Exactive Plus (Thermo Scientific). Peptides were separated by a reverse phase column (Accucore RP‐MS 100 × 2.1 mm, particle size, 2.6 μm). The column was maintained at a constant temperature of 40°C at a flow rate of 0.200 mL/min. Mobile phases A and B were water and acetonitrile, respectively, both acidified using 0.1% formic acid. The analysis was performed using the following gradient: 0‐5 minutes from 2% to 5% B; 5‐55 minutes from 5% to 30% B; 55‐61 minutes from 30% to 90% B and held for 1 minute, at 62.1 minutes the percentage of B was set to the initial condition of the run at 2% and held for 8 minutes in order to re‐equilibrate the column, for a total run time of 70 minutes. The mass spectrometry analysis was performed in positive ion mode. The electrospray ionization source was used with a voltage of 2.8 kV. The capillary temperature, sheath gas flow, auxiliary gas, and spare gas flow were set at 325°C, 45 arbitrary unit (arb), 10 arb, and 2 arb respectively. S‐lens was set at 70 radio frequency. For the acquisition of spectra, a data‐dependent (ddMS2) top 10 scan mode was used. Survey full‐scan mass spectrometry spectra (mass range m/z 381 to 1581) were acquired with resolution R = 70 000 and automatic gain control target 3 × 106. The MS/MS fragmentation was performed using high‐energy c‐trap dissociation (HCD) with resolution R = 35 000 and AGC target 1 × 106. The normalized collision energy (NCE) was set to 30. The injection volume was 3 μL. The mass spectra analysis was carried out using MaxQuant software (version 1.6.14). MaxQuant parameters were set as follows: trypsin was selected for enzyme specificity; the search parameters were fixed to an initial precursor ion tolerance of 10 ppm and MS/MS tolerance at 20 ppm; carbamidomethylation was set as fixed modification, whereas oxidation was set as variable modification. The maximum missed cleavages were set to 2. Andromeda search engine searched the spectra in MaxQuant against the National Center for Biotechnology Information *Felis catus* protein database (58 202 entries). Label‐free quantification was performed including a match between run option with the following parameters: protein and peptide false discovery rate set to 0.01, quantification based on the extracted ion chromatograms with a minimum ratio count of 1, and minimum required peptide length set to 7 amino acids.

### 2.7 Histology

Kidney, liver, and spleen samples were fixed in 10% neutral buffered formalin, embedded in paraffin, and cut to yield serial 4 to 5 μm thick sections. The sections were routinely stained with hematoxylin and eosin, periodic acid‐Schiff, Masson's trichrome, and periodic acid‐methenamine silver. Congo red staining was performed on 8‐ to 10‐μm thick sections. In kidney samples, amyloid deposits were classified based on their localization as cortical or medullary, and as glomerular, interstitial, or vascular. The amounts of deposits were scored from 0 to 2, as follows: 0 if fibrils were absent, 1 if mild focal deposits were detected, and 2 if moderate to severe focal deposits or if multifocal deposits were present. For each kidney specimen, the following data was evaluated using the longitudinal kidney section: mineralization of Bowman's capsule, wrinkling and thickening of the glomerular basement membrane, type and severity of glomerular hypercellularity, increase in glomerular matrix, glomerulosclerosis, severity and extension of interstitial fibrosis, tubular degeneration and atrophy, assessment of tubular basement membranes, as well as type, distribution, and severity of interstitial inflammation. Vascular lesions such as arteriolosclerosis and arteriolar mineralization also were assessed. All sections were independently examined by 2 observers (LC, SF) who were blinded to the medical records of the cats.

To characterize amyloidosis as AA, immunofluorescence was performed on kidney, spleen, and liver samples (Figure S1). 1% thioflavine S (T1892, Sigma‐Aldrich, Buchs, Switzerland) in double distilled H_2_O was used as a stain for amyloid aggregates, and nuclei were stained with 4′6‐diamidino‐2‐phenylindole. Polyclonal antibodies against cat A amyloid were added, followed by a rat anti‐mouse IgG conjugated to biotin (Cat#13‐4013‐85, ThermoFisher, Basel, Switzerland) and a streptavidin conjugated Alexa546 (s11225, Molecular Probes, Eugene, OR). Images were acquired using AxioImager A2 and AxioCam (Carl Zeiss, Jena, Germany).^16^

### 2.8 Statistical and bioinformatics analysis

Cats were divided into 2 groups (ie, cases with histological diagnosis of renal AA amyloidosis and cases without renal AA amyloidosis). Results of urine tests including USG, UPC ratio, SDS‐AGE profile, UAAC ratio, and proteomics analysis, as well as results of renal histology, including any renal lesion, glomerulosclerosis, fibrosis, and interstitial nephritis, were compared between groups to identify biomarkers that would help clinicians to noninvasively diagnose renal AA amyloidosis in cats with proteinuria. In cats with renal AA amyloidosis the concurrent presence of fibrils in the liver and spleen was compared between cases with score 1 vs score 2 and in cases with glomerular involvement vs without glomerular involvement. Unpaired *t*‐tests and Mann‐Whitney tests were used to compare USG, UPC ratio, and UAAC ratio, and Chi‐squared and Fisher's exact tests for SDS‐AGE profiles and renal histologic findings, using GraphPad Prism (version 5.0, GraphPad Software, San Diego, CA). Results of proteomics analysis were normalized for urine creatinine concentrations and compared using MaxQuant (version 1.6.14) and Marker View (Sciex, Concorde, Canada) software. Modulated proteins were analyzed using String (v.11.0) (http://string-db.org), which is a database of known and predicted protein‐protein interactions.^30^ Finally, in cats with renal AA amyloidosis, Fisher's exact test was used to compare the concurrent presence of amyloid fibrils in the liver and spleen between cats with kidney deposits score 1 vs score 2 and between cats with glomerular involvement vs without glomerular involvement to determine if more widespread amyloidosis was associated with higher renal score and glomerular involvement.

## 3 RESULTS

### 3.1 Animals

Twenty‐nine DSH cats with proteinuria were included, 19 with renal AA amyloidosis (65.5%) and 10 without renal AA amyloidosis (34.5%). No significant difference was found between cats with and without renal AA amyloidosis for sex, age, breed, and duration of stay in the shelter (Table 1). All cats were not recently vaccinated. Complete medical records were available for 17 of the 29 cats (58.6%): 12 with renal AA amyloidosis and 5 without (Table 2). All 12 cats with renal AA amyloidosis had CKD. According to IRIS guidelines, CKD was classified as IRIS stage I in 4 cats, II in 3, and IV in 5. Along with CKD, all cats with renal AA amyloidosis had ≥1 comorbidities: feline leukemia virus (FeLV) in 8 cats, feline immunodeficiency virus (FIV) in 3, feline infectious peritonitis (FIP) in 2, liver disease in 2 and ocular carcinoma, bone marrow aplasia, inflammatory bowel disease, and systemic feline calicivirus in 1 cat each. Causes of death in the 12 cats were CKD in 5, FIP in 2, liver disease in 2 and bone marrow aplasia, systemic calicivirus infection and ocular carcinoma in 1 each. In the 5 cats without renal AA amyloidosis, 2 had CKD, 1 FIP, 1 intestinal lymphoma and 1 bone marrow aplasia; these diseases also were the cause of death. According to IRIS guidelines, CKD was classified as IRIS stage III and IV in 2 cats. One of the 5 cats tested positive for FeLV antigen and another for FIV antibodies.

**TABLE 1:** Signalment in 19 cats with and in 10 cats without renal AA amyloidosis.

	AA amyloidosis cats	Non‐AA amyloidosis cats	*P*‐value
Spayed female	10 (52.6%)	5 (50%)	–
Neutered male	9 (47.4%)	5 (50%)	–
Age in years (range)	7 (1‐13)	6 (3‐17)	.53
Breed	19 DSH (100%)	10 DSH (100%)	–
Duration of stay in the shelter in months (range)	46 (5‐120)	25 (6‐204)	.44

Abbreviation: DSH, domestic shorthair.

**TABLE 2:** Clinicopathological features in 19 cats with and in 10 cats without renal AA amyloidosis.

	AA amyloidosis cats	Non‐AA amyloidosis
Cats with medical records	12 (63.2%)	5 (50%)
CKD	12 (63.2%)	2 (20%)
IRIS stage (I, II, III, IV)	4, 3, 0, 5	0, 0, 0, 2
Other diseases	–	Lymphoma, medullary aplasia, FIP
Concurrent diseases in CKD cats	FIP (2), liver disease (2), ocular carcinoma, systemic calicivirosis, inflammatory bowel disease, medullary aplasia	–
Retroviruses tested	13 (68.4%)	5 (50%)
FeLV	8 (61.5%)	1 (20%)
Non‐FeLV	5 (38.5%)	4 (80%)
FIV	3 (23.1%)	1 (20%)
Non‐FIV	10 (76.9%)	4 (80%)

Abbreviations: CKD, chronic kidney disease; FeLV, feline leukemia virus; FIP, feline infectious peritonitis; FIV, feline immunodeficiency virus; IRIS, international renal society.

### 3.2 Histology

Of the 19 cats with renal AA amyloidosis, 15 had concurrent hepatic and splenic involvement, whereas 2 had only the spleen involved (Table 3). Amyloidosis AA in the kidney was scored 1 in 8 cats and 2 in 11; score 2 was associated with AA amyloidosis concurrently involving the liver and spleen (*P* = .02).

**TABLE 3:** Histologic features in 19 cats with and in 10 cats without renal AA amyloidosis.

AA amyloidosis score in the kidney and distribution to organs (number of cats and %)			
Renal score 1	Renal score 2	Liver	Spleen
8 (42.1%)	11 (57.9%)	15 (78.9%)	17 (89.5%)

Renal AA amyloidosis localization			
Cortical	Medullary	Cortical and medullary	Vascular
3 (15.8%)	4 (21.1%)	12 (63.2%)	10 (52.6%)

Glomerular compartment involved		
Cortical	Medullary	Cortical and medullary
3 (100%)	0 (0%)	11 (91.7%)

Tubulointerstitial compartment involved		
Cortical	Medullary	Cortical and medullary
0 (0%)	4 (100%)	12 (100%)

In the kidney, AA amyloid was detected in the cortex in 15 of 19 cats (78.9%) and in the medulla in 16 (84.2%). Overall, amyloid was concomitantly observed in the cortex and medulla in 12 cats, and only in the cortex and only the medulla in 3 and 4 cats, respectively.

Amyloid deposits in the glomeruli were detected in 14 of 19 cats (73.7%); 11 also had fibrils observed in the medulla, in addition to the cortex. Of note, cats with glomerular amyloid deposits, compared to those without glomerular involvement, more frequently had concurrent fibrils in the liver and spleen (*P* = .04). Interstitial amyloid deposits were detected in 16 of 19 cats (84.2%); all had fibrils in the medulla and 9 also in the cortex. Ten of 19 cats (52.6%) had vascular amyloid deposits. Overall, fibrils were concurrently observed in the glomeruli, interstitium, and vessels in 9 cats, concurrently observed in the glomeruli and interstitium in 2, concurrently observed in the interstitium and vessels in 1, only in the interstitium in 4, and only in the glomeruli in 3.

**FIGURE 1:**
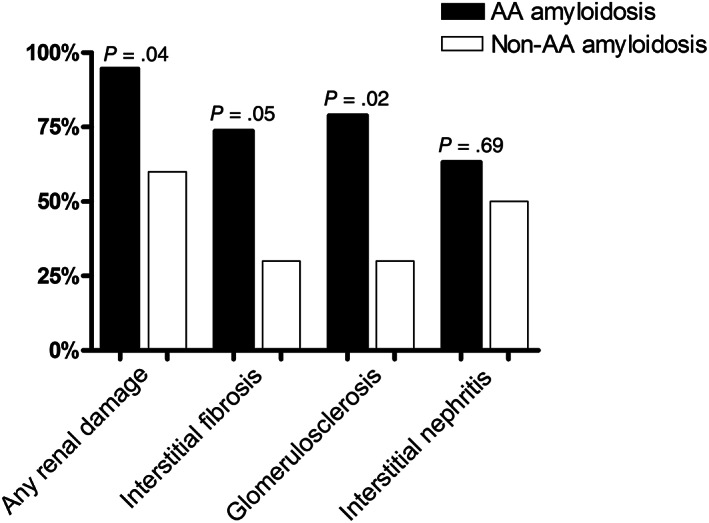
Prevalence of renal histologic lesions in the kidney of 19 cats with and 10 cats without renal AA amyloidosis.

Renal AA amyloidosis was associated with the presence of any microscopic lesions of the kidney (*P* = .04; Figure 1). Specifically, it was associated with glomerulosclerosis (*P* = .02) and interstitial fibrosis (*P* = .05). In particular, glomerulosclerosis was detected in 78.9% of cats with renal AA amyloidosis and in 30% of those with renal disease but without renal AA amyloidosis. Similarly, interstitial fibrosis was detected in 73.7% of the former and only in 30% of the latter. Conversely, interstitial nephritis was not associated with renal AA amyloidosis (*P* = .69).

### 3.3 USG, UPC ratio, SDS‐AGE, and UACC ratio

Median time from urine collection to death was 0 days (0‐19) in cats with renal AA amyloidosis and 0 days (0‐17) in those without renal AA amyloidosis. The USG did not differ between cats with and without renal AA amyloidosis (median, 1.016; range, 1.010‐1.050 vs 1.017; 1.010‐1.060; *P* = .35). Urine culture was performed in 7 cats with renal AA amyloidosis and in 4 without, respectively. Results were negative in all but 1 cat with renal AA amyloidosis (*Enterococcus* spp.).

**FIGURE 2:**
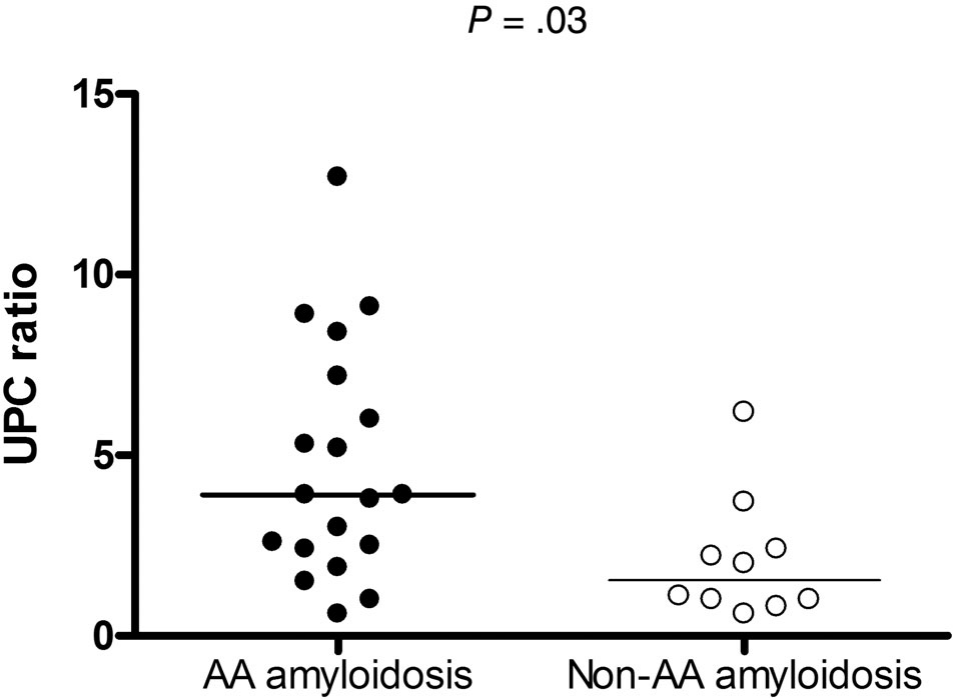
UPC ratio in 19 cats with and in 10 cats without renal AA amyloidosis. Solid lines represent the median value.

Cats with renal AA amyloidosis had higher UPC ratios compared to those without (median, 3.9; range, 0.6‐12.7 vs 1.5; 0.6‐3.1; *P* = .03; Figure 2).

The SDS‐AGE was performed in all cats, except for 1 cat without renal AA amyloidosis because the bands were not clearly visible. It identified HMW proteinuria in 89.5% of cats with renal AA amyloidosis and in 77.8% of those without renal AA amyloidosis (*P* = .57; Table 4). low molecular weight proteinuria was observed in 94.7% of cats with renal AA amyloidosis and in 66.6% of those without (*P* = .08). Overall, among cats with renal AA amyloidosis, proteinuria was mixed in 89.4%, glomerular in 5.3%, and tubular in 5.3%. Of cats without renal AA amyloidosis, proteinuria was mixed in 55.6%, glomerular in 11.2%, tubular in 11.2%, and no bands were observed in 22.2% (*P* = .45).

**TABLE 4:** SDS‐AGE in 19 cats with and in 9 cats without renal AA amyloidosis.

	AA amyloidosis cats	Non‐AA amyloidosis cats	*P*‐value
HMW proteinuria			
Absent	2 (10.5%)	2 (22.2%)	
Present	17 (89.5%)	9 (77.8%)	.57
Absent/mild	7 (36.8%)	5 (55.6%)	
Moderate/severe	12 (63.2%)	4 (44.4%%)	.43
LMW proteinuria			
Absent	1 (5.3%)	3 (33.3%)	
Present	18 (94.7%)	6 (66.6%)	.08
Absent/mild	8 (42.1%)	5 (55.5%)	
Moderate/severe	11 (57.9%)	4 (44.4%)	.69
Proteinuria classification			
Mixed	17 (89.4%)	5 (55.6%)	
Glomerular	1 (5.3%)	1 (11.1%)	.67
Tubular	1 (5.3%)	1 (11.1%)	
Absent	0 (0%)	1 (22.2%)	

Abbreviations: HMW, high molecular weight; LMW, low molecular weight; SDS‐AGE, sodium dodecyl sulfate‐agarose gel electrophores.

**FIGURE 3:**
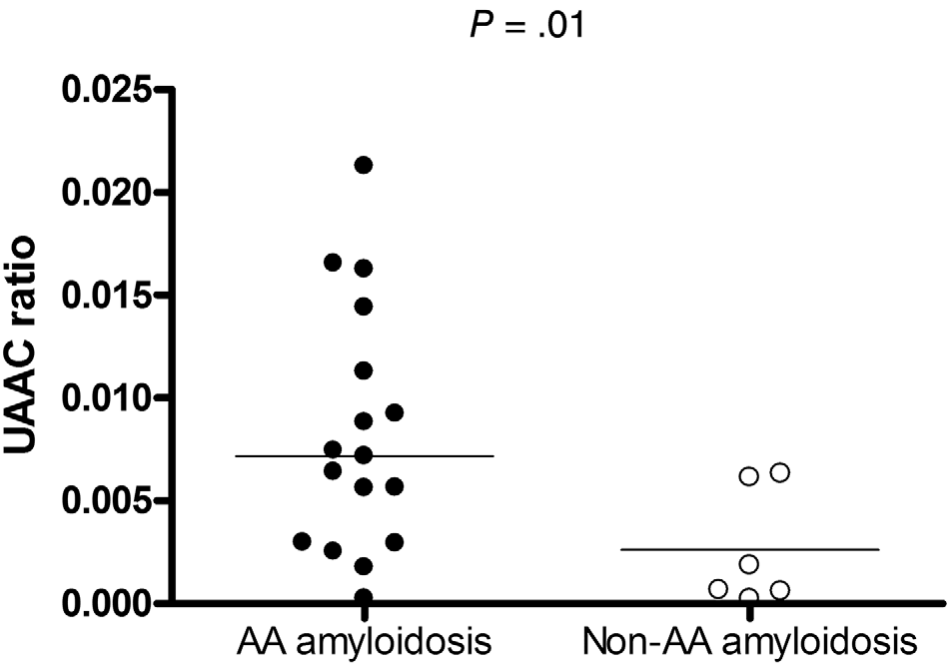
UAAC ratio in 17 cats with and in 6 cats without renal AA amyloidosis. Solid lines represent the median value.

The UAAC ratio was measured in 17 cats with renal AA amyloidosis and in 6 without. The former had a higher UAAC ratio than the latter (median, 7.18 × 10^−3^; range, 0.23 × 10^−3^‐21.29 × 10^−3^ vs 1.26 × 10^−3^; 0.21 × 10^−3^‐6.33 × 10^−3^; *P* = .04; Figure 3).

### 3.4 LC‐MS of urine proteins

**FIGURE 4:**
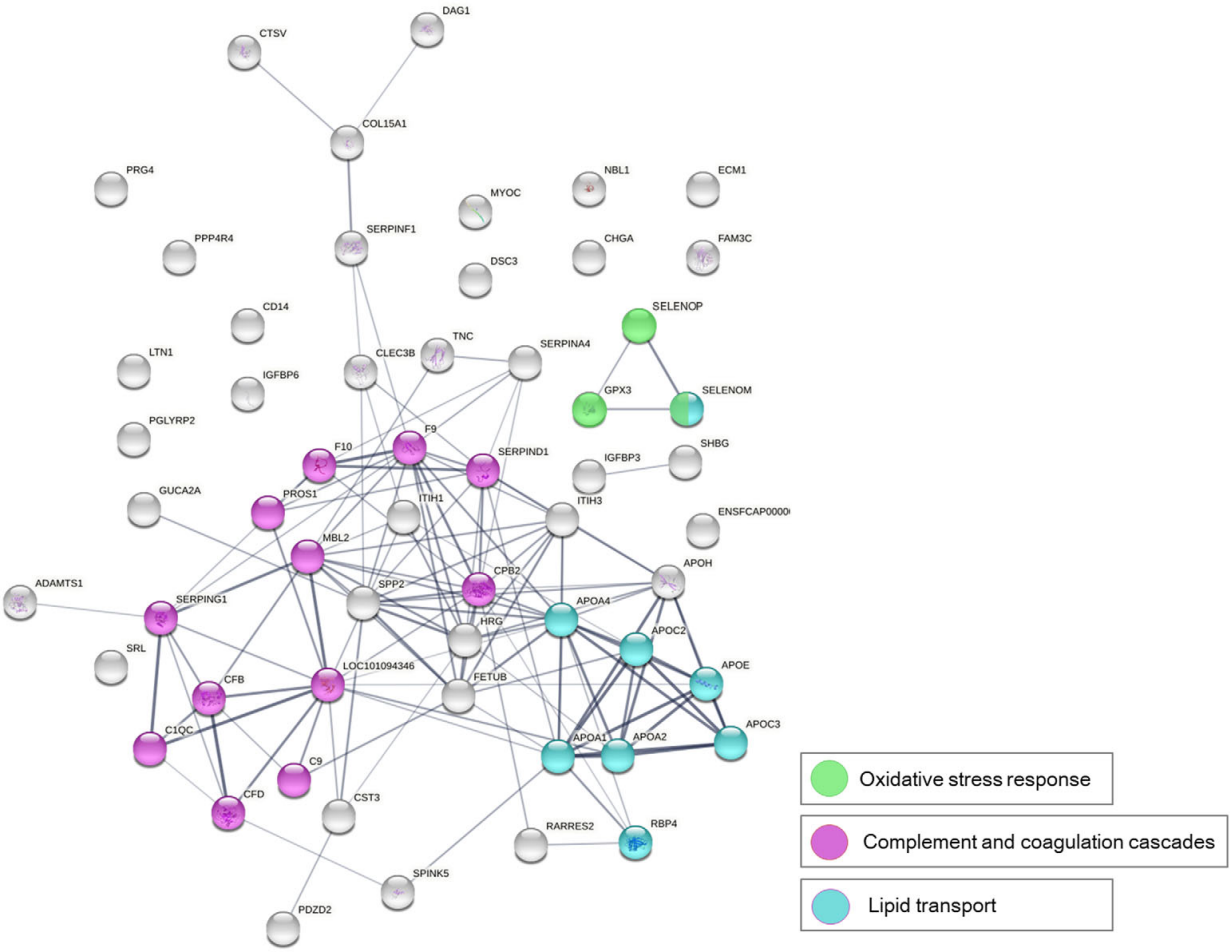
Network and pathway analysis of overexpressed proteins in urine of cats with renal AA amyloidosis compared to cats without renal AA amyloidosis. Proteins are mainly involved in oxidative stress (green), lipid transport (light blue), and complement system and coagulation cascade pathway (pink). Proteins are indicated by nodes labeled with the gene symbol. All their interactions were derived from high‐throughput lab experiments and previous knowledge in curated databases at high level of confidence (Sources: experiments, databases; gray dark lines for score ≥0.90). Line thickness indicates the strength of data support).

Proteomics analysis identified 2057 urine proteins, including 63 with higher and 62 with lower amounts in cats with renal AA amyloidosis compared to those without renal AA amyloidosis. Higher abundant proteins were mainly involved in oxidative stress, lipid transport, complement system, and coagulation cascade pathways (Figure 4; Table 5). Regarding oxidative stress proteins, those more concentrated in cats with renal AA amyloidosis compared to cats without renal AA amyloidosis were glutathione peroxidase (median, 2.82 × 10^7^; range, 5.25 × 10^6^‐4.98 × 10^7^ vs 1.12 × 10^7^; 2.22 × 10^6^‐3.26 × 10^7^; *P* < .001) and selenoprotein P (median, 4.99 × 10^6^; range, 2.47 × 10^6^‐2.99 × 10^6^ vs 2.29 × 10^6^; 7.31 × 10^5^‐4.45 × 10^6^; *P* < .001). Among complement and coagulation cascade proteins, complement C4‐A (median, 3.68 × 10^8^; range, 1.35 × 10^8^‐7.27 × 10^8^ vs 1.65 × 10^8^; 3.70 × 10^7^‐4.18 × 10^8^; *P* = .001), complement C9 (median, 1.14 × 10^8^; range, 3.83 × 10^7^‐2.69 × 10^8^ vs 5.68 × 10^7^; 8.25 × 10^6^‐1.12 × 10^8^; *P* = .005) and complement factor D (median, 8.59 × 10^8^; range = 1.05 × 10^7^‐3.4 × 10^9^ vs 6.31 × 10^7^; 1.26 × 10^6^‐6.01 × 10^8^; *P* = .008) were higher in cats with renal AA amyloidosis compared to cats without AA amyloidosis. Among lipid transport proteins, different apolipoproteins were excreted more abundantly in cats with renal AA amyloidosis compared to unaffected cats. In particular, Apo C‐III (median, 1.38 × 10^7^; range, 1.85 × 10^5^‐5.29 × 10^7^ vs 1.76 × 10^6^; 0 × 10^0^‐1.38 × 10^7^; *P* = .007), Apo E (median, 9.13 × 10^7^; range, 1.58 × 10^7^‐7.14 × 10^8^ vs 7.75 × 10^6^; 5.36 × 10^5^‐2.27 × 10^7^; *P* = .02) and Apo A‐I (median, 1.86 × 10^9^; range, 9.15 × 10^7^‐8.72 × 10^9^ vs 4.07 × 10^8^; 2.93 × 10^7^‐3.69 × 10^9^; *P* = .023). All the reported values are expressed as protein relative abundance.

**TABLE 5:** Upregulated proteins in 19 cats with and in 10 cats without renal AA amyloidosis, divided in different groups based on protein physical and functional class.

	AA amyloidosis cats	Non‐AA amyloidosis cats	*P*‐value
Lipid transport			
Apolipoprotein C‐III	1.38 × 10^7^ (1.85 × 10^5^‐5.29 × 10^7^)	1.76 × 10^6^ (0.0‐1.38 × 10^7^)	.01
Apolipoprotein E	9.13 × 10^7^ (1.58 × 10^7^‐7.14 × 10^8^)	7.75 × 10^6^ (5.36 × 10^5^‐2.27 × 10^7^)	.02
Rethinol binding protein 4 precursor	1.69 × 10^9^ (7.92 × 10^7^‐1.34 × 10^10^)	2.97 × 10^8^ (6.44 × 10^6^‐1.44 × 10^9^)	.02
Apolipoprotein A‐I	1.86 × 10^9^ (9.15 × 10^7^‐8.72 × 10^9^)	4.07 × 10^8^ (2.93 × 10^7^‐3.69 × 10^9^)	.02
Apolipoportein A‐II	1.87 × 10^6^ (0.00 × 10^0^‐8.15 × 10^6^)	9.10 × 10^5^ (9.50 × 10^4^‐2.03 × 10^6^)	.03
Apolipoprotein A‐IV	8.96 × 10^7^ (1.49 × 10^6^‐5.96 × 10^8^)	4.86 × 10^6^ (3.41 × 10^5^‐4.48 × 10^7^)	.03
Selenoprotein M	0.00 (0.00‐1.19 × 10^6^)	0.00 (0.00‐7.58 × 10^4^)	.03
Apolipoportein C‐II	3.64 × 10^5^ (0.00‐6.04 × 10^6^)	0.00 (0.00‐6.75 × 10^5^)	.05
Complement and coagulation cascade			
Complement C4	3.68 × 10^8^ (1.35 × 10^8^‐7.26 × 10^8^)	1.65 × 10^8^ (3.70 × 10^7^‐4.71 × 10^8^)	.001
Coagulation factor IX	1.32 × 10^7^ (2.21 × 10^6^‐2.98 × 10^7^)	2.37 × 10^6^ (0.00‐9.66 × 10^6^)	.001
Plasma protease C1	5.82 × 10^7^ (1.45 × 10^7^‐1.22 × 10^8^)	1.17 × 10^7^ (7.56 × 10^5^‐5.04 × 10^7^)	.001
Complement C1	6.80 × 10^6^ (0.00‐3.31 × 10^6^)	0.00 (0.00‐4.90 × 10^5^)	.01
Heparin cofactor 2	4.34 × 10^7^ (9.71 × 10^6^‐1.22 × 10^8^)	2.30 × 10^7^ (2.06 × 10^6^‐4.45 × 10^7^)	.02
Coagulation factor X	3.05 × 10^6^ (3.65 × 10^5^‐3.97 × 10^7^)	4.75 × 10^5^ (1.19 × 10^5^‐2.90 × 10^6^)	.04
Carboxypeptidase B2	2.25 × 10^7^ (6.27 × 10^6^‐1.25^8^)	1.36 × 10^7^ (4.40 × 10^6^‐2.25 × 10^7^)	.04
Complement factor B	3.47 × 10^8^ (1.55 × 10^8^‐1.09 × 10^9^)	1.57 × 10^8^ (3.51 × 10^5^‐6.40 × 10^8^)	.04
Mannose binding protein C	3.37 × 10^6^ (2.13 × 10^5^‐3.86 × 10^6^)	4.67 × 10^5^ (0.00‐4.15 × 10^6^)	.05
Vitamin K dependent	2.48 × 10^6^ (2.80 × 10^5^‐1.10 × 10^7^)	1.28 × 10^6^ (0.00‐2.70 × 10^6^)	.05
Oxidative stress response			
Glutathione peroxidase	2.82 × 10^7^ (5.24 × 10^6^‐4.98 × 10^7^)	1.12 × 10^7^ (2.22 × 10^6^‐3.06 × 10^7^)	.001
Selenoprotein P	5.00 × 10^6^ (2.47 × 10^6^‐1.02 × 10^7^)	2.29 × 10^6^ (4.45 × 10^6^‐7.31 × 10^5^9	.001
Selenoprotein M	0.00 (1.19 × 10^6^ × 10^0^)	0.00 (0.00‐7.58 × 10^4^)	.03

Among the proteins that were less abundant in cats with renal AA amyloidosis compared to those without, no predominant pattern was observed.

## 4 DISCUSSION

In our study, cats with proteinuria and renal AA amyloidosis had higher UPC and UAAC ratios compared to cats with proteinuria but without renal AA amyloidosis. Urine SDS‐AGE did not differentiate cats with and without renal AA amyloidosis. The LC‐MS identified increased urine excretion of proteins involved in the oxidative stress response, lipid transport, complement system, and coagulation cascade pathways in cats with renal AA amyloidosis. Regarding histology, renal AA amyloidosis was associated with glomerulosclerosis and renal fibrosis, but not interstitial nephritis. Among cats with renal AA amyloidosis, those with higher amounts of fibrils or with fibrils in glomeruli more frequently had AA amyloidosis concurrently affecting the liver and spleen.

Similar to a recent study, approximately two‐thirds of shelter cats with renal AA amyloidosis had fibril deposition in both the cortex and medulla, whereas in Abyssinian cats with familial AA amyloidosis deposits were predominantly observed in the medulla. It is likely that the pathogenesis of the disease is different in DSH than in Abyssinian cats, leading to differences in parenchymal distribution of fibrils.^8^, ^9^, ^10^, ^20^

In our study, cats with renal AA amyloidosis and higher amounts of fibrils were more likely to have deposits involving the liver and the spleen, suggesting possibly that marked deposition in the kidneys represents an advanced stage of the disease with multiorgan involvement. Furthermore, an association was observed between the presence of renal AA amyloidosis and renal lesions in cats, and in particular glomerulosclerosis and fibrosis. In previous studies in dogs and cats with familial AA amyloidosis and kidney involvement, several lesions were observed, including glomerulosclerosis, fibrosis, interstitial nephritis, tubular atrophy, and obsolescent glomeruli.^8^, ^31^, ^32^ Nonetheless, in none of these studies was an association with AA amyloidosis explored. Based on our results, and despite the limited number of cases included in our case series, strategies to prevent and treat AA amyloidosis might be recommended in affected cats to avoid or slow the progression of renal disease. The pathogenesis of amyloid‐induced renal damage has not been clearly elucidated. In a study of humans with renal AA amyloidosis, it was hypothesized that glomerular deposition of amyloid fibrils induces structural lesions that decrease the filtration rate and cause proteinuria, with the latter promoting glomerulosclerosis and fibrosis.^33^ Based on in vitro studies, amyloid precursor proteins or their folding intermediates can cause cell toxicity by damaging lipid membranes.^34^, ^35^ Whether a similar situation occurs in cat is unknown.

Cats with proteinuria and renal AA amyloidosis had higher UPC ratios compared to shelter cats with proteinuria and without the protein misfolding disease but the overlap between affected and unaffected cats did not make it a useful marker to identify those with renal deposits. Hence, the presence of proteinuria in cats without AA amyloidosis suggests that other tests are necessary to identify those with deposition of fibrils in the kidneys. In our case series, it was observed that 40% of cats without AA amyloidosis had no evidence of renal damage based on optical microscopy. It is probable that lesions would have been noticed in the latter cats using electron microscopy. Alternatively, lesions were multifocal and not identified, even if renal samples were collected at necropsy and large portions of tissue were examined. Finally, it cannot be excluded that prerenal or postrenal proteinuria was present in some cases. Of note, 1 cat with amyloidosis had a positive bacterial culture and markedly increased UPC ratio (5.2). However, the cat had normal urine sediment and showed no clinical signs of urinary tract infection. Hence, it is possible that bacterial contamination had occurred.

Mixed proteinuria was observed in Abyssinian cats with familial amyloidosis by SDS‐PAGE.^22^ In our study, mixed proteinuria was identified in most of cats with and without renal AA amyloidosis. Accordingly, SDS‐AGE is not helpful for diagnostic purposes in a shelter setting.

The UAAC ratio was higher in cats with renal AA amyloidosis despite some overlap with unaffected cats. Nonetheless, in affected cats the median UAAC ratio was higher than the maximum value documented in cats without AA amyloidosis, suggesting that a markedly increased UAAC ratio is compatible with renal AA amyloidosis, similar to a previous study performed in Abyssinian cats with the disease.^22^

In cats, oxidative stress is involved in different metabolic conditions such as diabetes mellitus, obesity, and chronic kidney disease.^36^, ^37^, ^38^ N^ε^‐carboxymethyllysine, an advanced glycation end‐product produced during protein oxidation, has been detected in the renal tissue of humans affected by AA amyloidosis with kidney involvement.^39^, ^40^ In our case series, glutathione peroxidase, selenoprotein P, and selenoprotein M were excreted in urine of cats with renal AA amyloidosis in larger amounts, suggesting a link between oxidative damage and the protein misfolding disease. It cannot be excluded that oxidative stress was partly caused by CKD, regardless of AA amyloidosis. Regardless of the cause, antioxidant treatment should be further evaluated in shelter cats with renal AA amyloidosis.

In human medicine, AA amyloidosis is associated with inflammatory diseases such as rheumatoid arthritis.^33^, ^41^ A permissive role of inflammatory diseases for the deposition of amyloid fibrils has been hypothesized in shelter cats.^19^ We observed that complement and coagulation cascade proteins were increased in the urine of cats with renal AA amyloidosis. Hence, our results suggest activation of some inflammatory pathways also may be associated with the pathophysiology of the disease in cats.

In addition, cats with renal AA amyloidosis had higher urine excretion of several lipid transport proteins, and in particular apolipoproteins (C‐III, E, and A‐I). Apolipoproteins are involved in transport of lipids and metabolism of lipoproteins and circulate bound to the latter.^42^, ^43^ During the inflammatory response, serum concentration of the acute phase protein SAA increases, and SAA binds to apolipoproteins displacing Apo A‐I.^44^ The bond between SAA and apolipoproteins occurs through the SAA N‐terminal fragment and prevents SAA misfolding because the unbound SAA is labile and prone to conformational change.^45^, ^46^, ^47^ Of note, apolipoproteins have been observed in kidneys from humans, cats, and island foxes with renal AA amyloidosis, including apolipoproteins A‐I, A‐IV, and E.^26^, ^27^, ^48^ Thus, they also might play an important role in shelter cats with the disease.

Our study had some limitations because of its retrospective design. In particular, some cases had incomplete medical records and few urine samples. In addition, 1 cat with renal AA amyloidosis had concurrent bacteriuria. Because the urine sediment was negative and the cat did not show clinical signs, its relevance to our results was likely negligible.

In conclusion, renal AA amyloidosis in shelter cats is associated with renal damage as well as increased proteinuria. Clinicians should suspect renal AA amyloidosis in shelter cats with urine protein loss and increased urine excretion of SAA. Additional studies are needed to clarify the role of proteins related to oxidative stress, complement system, coagulation cascade, and, in particular, lipid transport in the urine of affected cats.

## CONFLICT OF INTEREST DECLARATION

Eric Zini serves as Associate Editor for the Journal of Veterinary Internal Medicine. He was not involved in the review of this manuscript. No other authors declare a conflict of interest.

## OFF‐LABEL ANTIMICROBIAL DECLARATION

Authors declare no off‐label use of antimicrobials.

## INSTITUTIONAL ANIMAL CARE AND USE COMMITTEE (IACUC) OR OTHER APPROVAL DECLARATION

Authors declare no IACUC or other approval was needed.

## HUMAN ETHICS APPROVAL DECLARATION

Authors declare human ethics approval was not needed for this study.

## Supporting information


**Figure S1.** Immunofluorescence staining for AA‐amyloidosis. Cat kidney tissue was stained with 4′,6‐diamidino‐2‐phenylindole (A, in blue). Thioflavin S was used to identify amyloid aggregates (B, in green) and anti‐AA IgG to confirm AA amyloidosis (C, in red). A merge of B and C was created to authenticate the specificity of the latter (D, in yellow).
