# Supplementary figures and images for: Renal amyloid‐A amyloidosis in cats: Characterization of proteinuria and biomarker discovery, and associations with kidney histology
Source: J Vet Intern Med. 2023 Nov 22;38(1):205–15. doi: 10.1111/jvim.16920 (PMC10800178; doi:10.1111/jvim.16920)

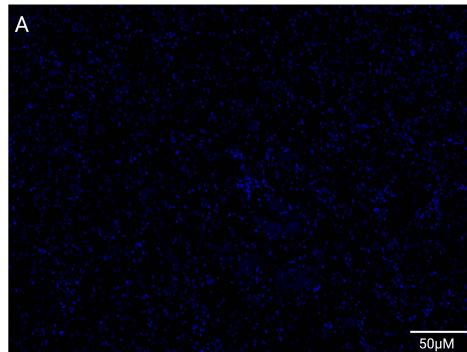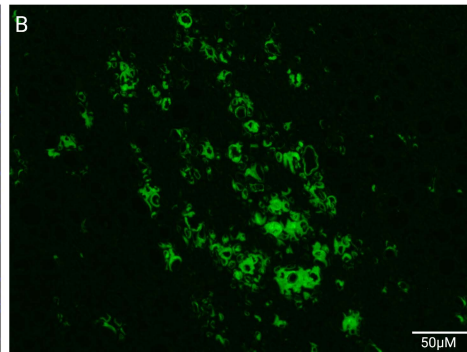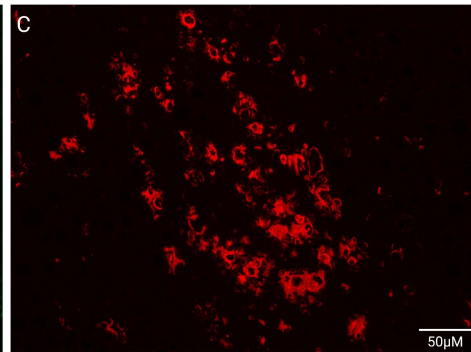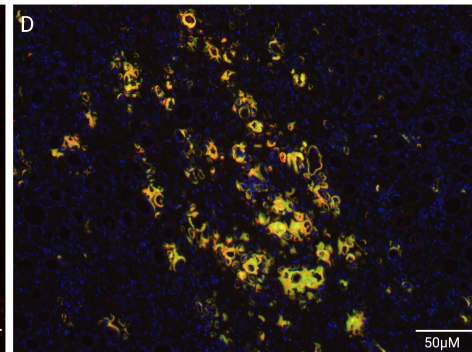

Supplement: Supplementary file 1 — Figure S1. Immunofluorescence staining for AA‐amyloidosis. Cat kidney tissue was stained with 4′,6‐diamidino‐2‐phenylindole (A, in blue). Thioflavin S was used to identify amyloid aggregates (B, in green) and anti‐AA IgG to confirm AA amyloidosis (C, in red). A merge of B and C was created to authenticate the specificity of the latter (D, in yellow). [file JVIM-38-205-s001.pdf]
